# Cannulated compression screw with versus without two K-wire fixation for treatment of scaphoid waist fracture nonunion

**DOI:** 10.1186/s13018-022-02975-z

**Published:** 2022-02-05

**Authors:** Xiaoran Zhang, Li Wang, Xuelin Ma, Fengyu Wang, Wenxu Duan, Xinzhong Shao

**Affiliations:** Department of Hand Surgery, The 3rd Hospital, Hebei Medical University, NO. 139 Ziqiang Road, Shijiazhuang, 050051 Hebei People’s Republic of China

**Keywords:** Scaphoid fracture nonunion, Tripod fixation, Anti-rotation K-wires, Comparative study

## Abstract

**Purpose:**

This study aims to introduce the “tripod” technique using cannulated compression screw and two anti-rotational K-wires for treatment of unstable scaphoid waist fracture nonunion, and to compare it with the single cannulated screw fixation technique in term of scaphoid union and surgical outcomes.

**Methods:**

It was a retrospective study. From January 2014 to March 2020, 103 consecutive patients with scaphoid waist fracture nonunion treated with the tripod fixation and bone grafting (*n* = 45) or with single cannulated compression screw and bone grafting (*n* = 58) were included. All the procedures were performed by the same hand surgery team, and autologous cortico-cancellous radius bone graft was used for bony reconstruction. The minimal follow-up period was 12 months. The union rate and the time to union, range of motion (ROM), grip strength, Visual Analogue Scale (VAS), Quick Disabilities of the Arm, Shoulder and Hand (DASH) score and modified Mayo Scores at the last visit were compared.

**Results:**

In tripod fixation group, bony union was achieved in all patients at the mean of 14.8 ± 3.8 weeks, while in the single cannulated screw fixation group the bony union rate was 94.8% (55/58) and the time to union was 17.6 ± 3.6 weeks. The difference of time to union was statistically significant (*p* = 0.027), but not for bony union rate (*p* = 0.122). At the last visit, no significant difference was found with respect to any clinical and radiographic outcome measures (all *p* > 0.05). The overall rate of complications was not significantly different between two groups (15.6% vs 10.3%, *p* = 0.430).

**Conclusions:**

Tripod fixation technique with headless compression screw and two K-wires is a safe and effective technique for treatment of scaphoid nonunion fixation and can be considered to use in practice, especially for those potentially rotationally unstable cases.

## Introduction

The fracture of the scaphoid is a common injury, accounting for 60–80% of all the carpal fractures and over 80% of the fractures occur in the waist [1–3]. Given the special anatomic location that the scaphoid serves a major connection in the proximal row of the carpus, it has an important role in the wrist function. Consequently, the nonunion of scaphoid fracture arising from none or inappropriate treatment can compromise the wrist function or even leave the long-term sequela. It is reported that, the risk of nonunion following scaphoid fracture is 5% roughly, and can be up to 15% in selected complex cases, e.g. open fracture, multiple fracture, high body mass index, smoking and alcoholism [4, 5].

Scaphoid waist fracture nonunion is still a challenge to hand surgeons, often requiring surgical management involving the combination of bone graft and internal fixation to restore the carpal alignment and length. Cannulated screw fixation has been shown to be the “gold standard” for scaphoid nonunion, but the demonstrated inability to provide sufficient stability might make it an imperfect method [6–8]. Over the last decade, two Herbert screw is favourably applied in practice duo to the biomechanical advantage, especially against rotation [6], but is technically more demanding and thus requires steep learning curve [9]. Besides, two Herbert screw fixation can occupy more space that decreases the amount of bone graft that can be placed, and affect the tenuous blood supply which is adverse to bone healing. Plate fixation is another currently used surgical choice [10–12], which can provide greater biomechanical rigidity relative to single screw fixation, but is technically more demanding and requires extended open dissection for plate placement, which may also compromise the bone healing.

In this study, we introduce the “tripod technique” using one headless compression screw and two de-rotational K-wires for treatment of unstable scaphoid waist nonunion, and to obtain comparative data to support its clinical use, we compare this technique with the “gold standard”, single cannulated screw fixation. We hypothesize that this “tripod” technique can increase the union rate and accelerate union of scaphoid waist nonunion.

## Methods

This was a retrospective observational study, approved by the ethics committee of The Third Hospital of Hebei Medical University. Written informed consent was obtained from each participant before its commencement. Between January 2014 and March 2020, 103 consecutive patients with scaphoid waist fracture nonunion treated with open reduction, cortico-cancellous radius bone grafting and internal fixation with either a single Herbert screw or a Herbert screw combined with two anti-rotational K-wires, were included for data analysis.

The inclusion criteria were: patient age of 18 years or older, scaphoid waist fracture not uniting more than 6 months since the initial injury, treatment by either technique, and the minimum 1-year follow-up assessments. The exclusion criteria were: bilateral scaphoid fractures nonunion, concomitant ipsilateral upper extremity fracture, previous surgery in scaphoid or upper extremities, scaphoid nonunion advanced collapse (SNAC) waist, scaphoid nonunion with avascular necrosis (AVN), radioscaphoid arthritis, or missing follow-up assessments.

## Surgical technique

### “Tripod” fixation

Operation was performed under regional anesthesia with a brachial plexus block using an upper-arm tourniquet. A radial approach was used as previously described by our team [13]. A longitudinal incision of 3-4 cm in length was made on the connection between the styloid process of the radius and the trapezium. The skin and subcutaneous tissue were cut, the cephalic vein and the superficial branch of the radial nerve were separated and retracted dorsally. The musculi abductor pollicis longus and extensor pollicis brevis tendon were pulled towards the palm side, and the joint capsule was cut longitudinally to expose the fractured end when wrist was at ulnar deviation. The fibrous tissue and sclerotic bone were resected up to normal-looking bone, and cortical-cancellous bone was harvested from the dorsal styloid process of the radius for use. Cancellous bone was scraped and filled in the bone defect, and cortical-cancellous bone was filled in the fracture gap to restore the scaphoid height. C-arm image intensifier was used to confirm the reduction and the alignment. From the distal end of the scaphoid tubercle, three K-wires, 1.0-mm in diameter, were inserted along the long axis of the scaphoid, 45° ulnar and 45° dorsal (namely, double 45° direction) to the neutral plane, in a convergent manner. C-arm image intensifier was used to confirm the satisfactory placement of the K-wires. The one K-wire that was optimally placed (namely, the most accordant with the long axis of the scaphoid, close to the center, and perpendicular to the fracture line) as the guide wire to screw the Herbert screw and achieve compression at the fractured end. The three K-wires were pre-bent and then cut short as possible and attached to the surface of the bone, to reduce skin and soft tissue irrigation and cartilage damage and facilitate wrist range of motion.

### Cannulated screw fixation

The operative procedure used for single Herbert screw fixation was the same as that for the above technique, only without need of using the two anti-rotation K-wires. As well, the autogenous bone grafting was performed to fill the bone detect using cortico-cancellous harvested from distal radius.

### Postoperative management

Both groups used the same postoperative rehabilitation regimen. Short arm splint was applied for the first postoperative 8 weeks. Patients were encouraged to elevate the affected arm as much to reduce swelling, and to allow active finger motion and shoulder exercises immediately after operation. At two weeks, skin sutures were removed. After the short splint was removed, patients were permitted to do aggressive wrist exercises. After obtaining radiographic evidence of scaphoid union, patients were allowed for heavy activities, and at this time point the K-wires were removed.

### Follow-up

Until the radiographic bony union was confirmed by the senior orthopedic surgeon, patients were instructed to return to the outpatient visit at two-week interval; after bony union was achieved, every 3-month-interval visit was done until 12 months postoperatively. At each visit, 4 radiographic views of the wrist were taken, including standard post-anterior, lateral, 45° pronation oblique, and Stecher view (scaphoid ulnar deviation). Based on Clay criteria [14], absence of adverse features (e.g. a gap at nonunion site, lucency around or shifting of the implant, or displacement of the graft) in at least three of the four views was deemed as scaphoid union. When radiographic adverse features disappeared, CT scanning of the wrist along the longitudinal axis of the scaphoid was performed to confirm the union. The time to union was recorded when > 50% trabecular bridging across the nonunion site was demonstrated on CT scans. Adverse features remaining on the radiographs or less than 50% trabecular bridging on CT scans at postoperative 24 weeks was considered as nonunion [15].

### Clinical and radiographic outcome

The primary outcome was the union rate. The secondary outcomes were the time to union, wrist pain assessed by the visual analogue scale (VAS), range of motion (ROM) (% of the healthy side), grip strength (% of healthy side), the Quick Disabilities of the Arm, Shoulder and Hand (DASH) score, and the modified Mayo Scores at the last visit, the lateral intra-scaphoid angle (LISA) and the height length ration (HLR).

### Statistical analysis

The continuous variables were expressed with mean and standard deviation (SD), and their normality was explored by the Shapiro–Wilk test. Student-*t* test was used to examine the between-group difference for those normally distributed variables, and otherwise, Mann–Whitney U test was used.

The categorical variables were expressed with number and percentage, and the between-group difference was examined by Chi-square or Fisher’s exact test. A *p* value less than 0.05 was considered as statistically significant. All the analyses were performed by the SPSS Statistics Software version 25.0 (IBM corporation, Armonk, New York, USA).

## Results

Among the 103 eligible consecutive patients, there were 77 male patients and 26 female patients, with an average age of 35.1 years. Most of the injuries were involving the dominant wrist (79.6%) and the right wrist (72.8%). Fall was the most common injury mechanism causing the initial fracture (82.5%). About two thirds (68.0%) of the non-union cases were classified as fibrous nonunion, and 22.0% as sclerotic. The time from initial fracture to the index surgery for repair the nonunion was 13.5 months. As presented in Table 1, there were no significant differences observed between tripod fixation and single cannulated screw fixation group (all *p* > 0.05).

**Table 1:** Patient demographics and injury characteristics between two groups

Variable	Tripod fixation group (*n* = 45)	Single cannulated screw fixation group (*n* = 58)	*p*
Age (year)	35.9 ± 6.8	33.4 ± 6.1	0.357
Gender (%)			0.870
Male	34 (75.6)	43 (74.1%)	
Female	11 (24.4)	15 (25.9)	
Injured side (%)			0.732
Left	13 (28.9)	15 (25.9)	
Right	32 (71.1)	43 (74.1)	
Dominance side			
Dominant	35 (77.8)	47 (81.0)	0.684
Non-dominant	10 (22.2)	11 (19.0)	
Smoking status			0.863
Current	17 (37.8)	22 (37.9)	
Past	4 (8.9)	7 (12.1)	
Never	24 (53.3)	29 (50.0)	
Mechanism of injury (%)			0.813
Fall	37 (82.2)	48 (82.8)	
Twisting	4 (8.9)	7 (12.1)	
Punch	2 (4.4)	2 (3.4)	
Others or unknown	2 (4.4)	1 (1.7)	
Herbert classification			0.859
D1 (fibrous nonunion)	31 (68.9)	39 (67.2)	
D2 (sclerotic nonunion)	14 (31.1)	19 (32.8)	
Initial treatment			0.931
No treatment	9 (20.0)	12 (20.7)	
Splint or cast immobilization	36 (80.0)	46 (79.3)	
Time from initial injury to surgery (months)	13.2 ± 7.5	13.8 ± 8.1	0.682

In the tripod fixation group, bony union was achieved in all the cases, and the time to union was 14.8 ± 3.8 weeks; while in the single cannulated screw fixation group, the bony union rate was 94.8% (55/58), and the time to union was 17.6 ± 3.6 weeks. The difference of time to union was significantly different (*p* = 0.027), but for bony union was not, only being a trend (*p* = 0.122). No significant difference (*p* = 0.430) was observed for the overall rate of complications (7/45, 15.6% in the tripod fixation group vs 6/58, 10.3% in the single cannulated screw fixation group). The superficial surgical site infection occurred in 3 cases and 2 cases, complex regional pain syndrome in 1 and 2, the sensitive scar at graft site in 1 and 2, in the tripod group and single cannulated screw fixation group, respectively; the K-wire was removed in ahead of schedule in 2 case in the tripod fixation group, due to pin loosening or migration.

With respect to the clinical and radiographic outcomes, no significant difference was found for any one, as pain assessment on VAS (1.4 ± 1.7 vs 1.6 ± 2.0, *p* = 0.203), ROM % of healthy side (92.3 ± 7.6 vs 93.7 ± 7.4, *p* = 0.483), grip strength % of healthy side (94.7 ± 4.5 vs 94.1 ± 5.2, *p* = 0.923), Quick DASH score (19.3 ± 4.1 vs 17.9 ± 5.2, *p* = 0.317), the Mayo score (87.6 ± 10.6 vs 86.4 ± 11.3, *p* = 0.722), LISA (34.5° ± 12.1° vs 33.7 ± 14.2°, *p* = 0.790) or HLR (0.67 ± 0.08 vs 0.63 ± 0.08, *p* = 0.917) (Table 2).

**Table 2:** Comparisons of clinical and radiographic outcomes at the last visit between both groups

Variable	Tripod fixation group (*n* = 45)	Single cannulated screw fixation group (*n* = 58)	*p*
Union rate	45/45 (100%)	55/58 (94.8%)	0.122
Time to union	14.8 ± 3.8	17.6 ± 3.6	0.027*
Pain assessment on VAS (0–10)	1.4 ± 1.7	1.6 ± 2.0	0.203
ROM % of healthy side	92.3 ± 7.6	93.7 ± 7.4	0.483
Grip strength % of healthy side	94.7 ± 4.5	94.1 ± 5.2	0.923
Quick DASH score (0–100)	19.3 ± 4.1	17.9 ± 5.2	0.317
Mayo score (0–100)	87.6 ± 10.6	86.4 ± 11.3	0.722
LISA	34.5° ± 12.1°	33.7 ± 14.2°	0.790
HLR	0.67 ± 0.08	0.63 ± 0.08	0.917

VAS, visual analogue scale score; LISA, lateral intrascaphoid angle; HLR, height length ration; DASH, Disabilities of the Arm, Shoulder, and Hand

^*^Statistically signiant

**Fig. 1:**
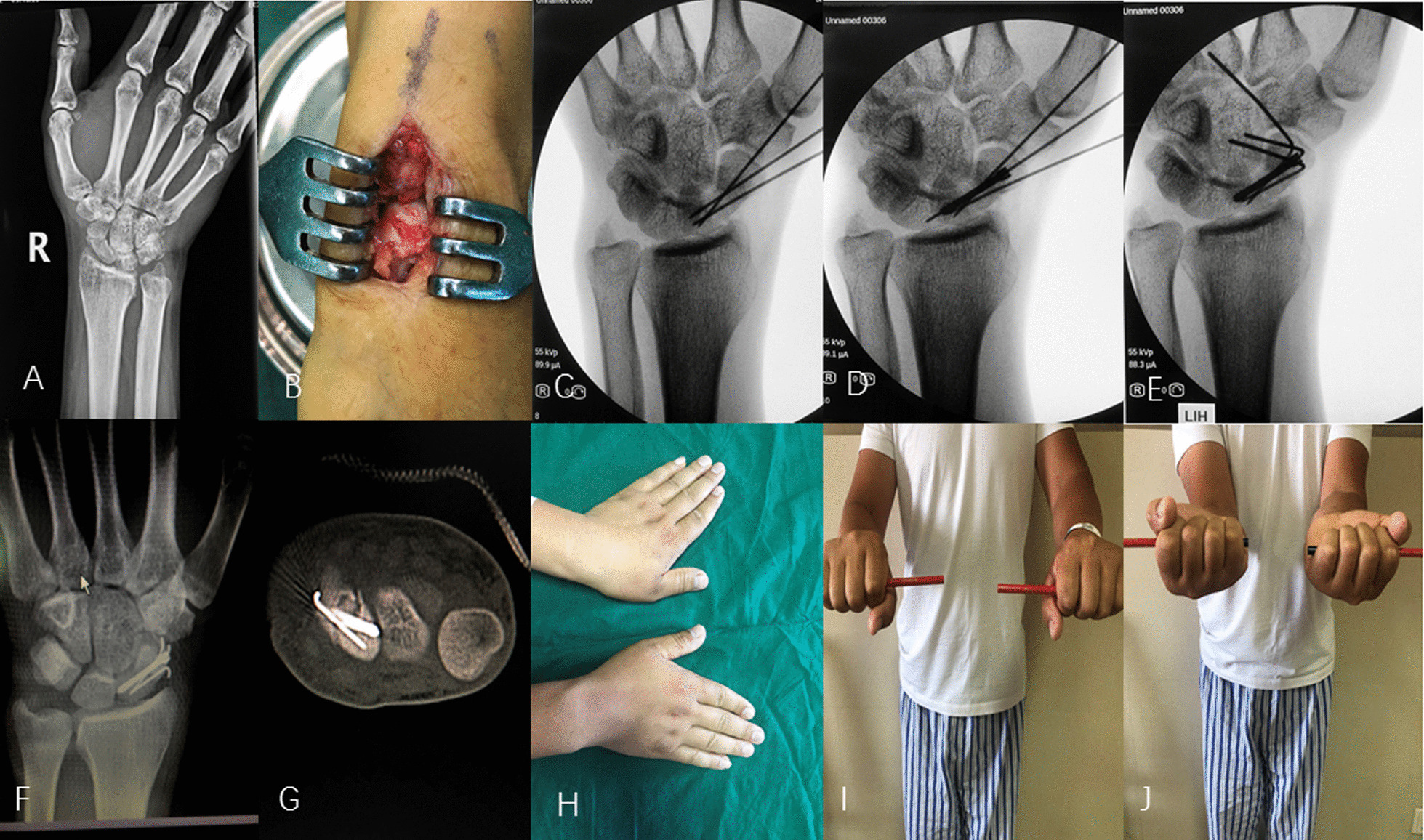
A 32-year young man sustained right scaphoid waist fracture nonunion 11 months after the initial fracture treated by conservative method. The **a** showed the nonunion at the scaphoid waist site. The **b** to **e** showed the operative process of tripod fixation using Herbert screw and two adjunctive K-wires. The **f**, **g** were the postoperative radiograph and the CT scan. **h** to **j** showed the excellent functional outcome at 12 months postoperatively

**Fig. 2:**
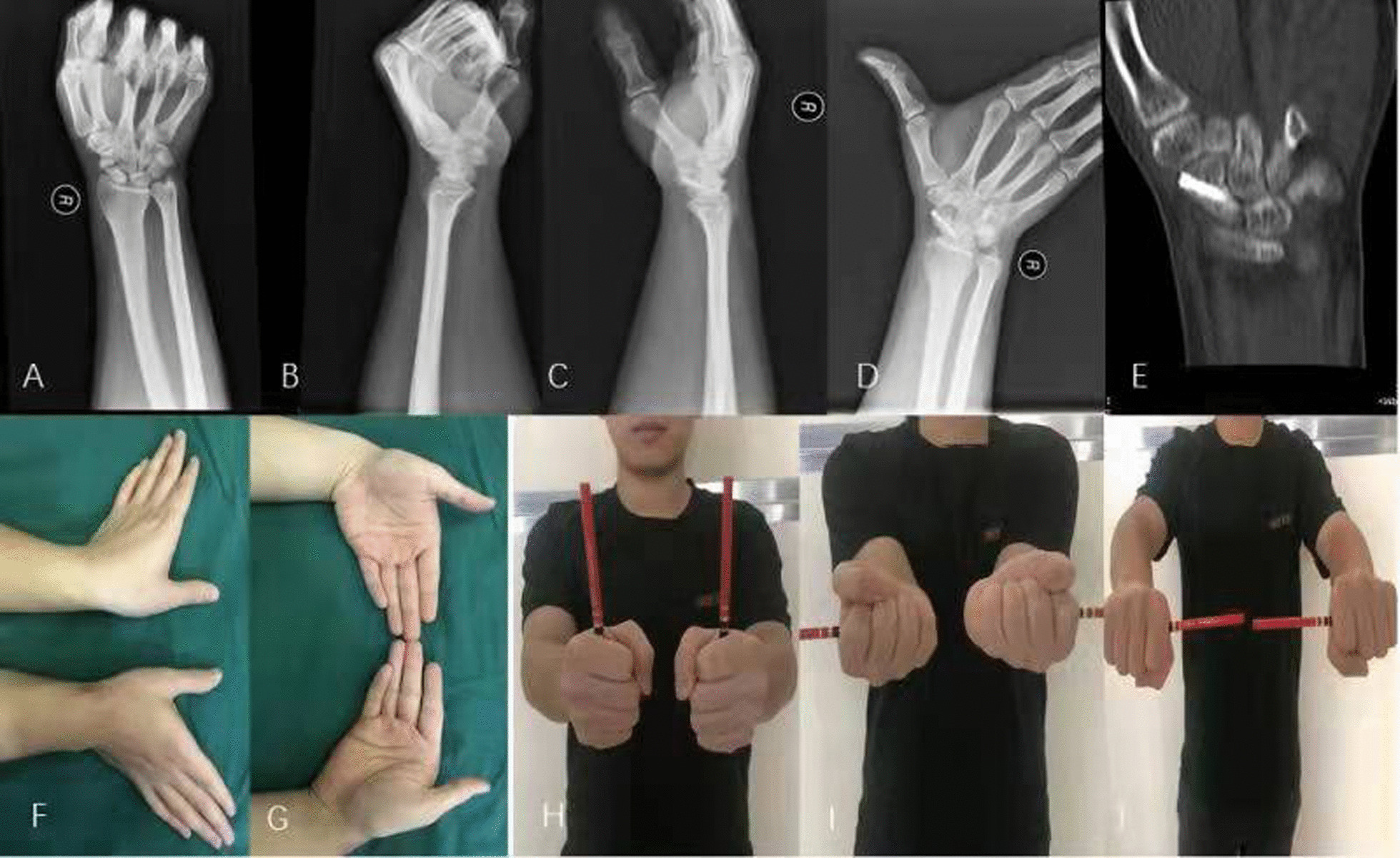
A 34-year young man sustained right scaphoid waist fracture nonunion 13 months after the initial fracture treated conservatively. The **a**, **b** sowed the nonunion at the waist. The **c** to **e** showed it was treated by single Herbert screw fixation. The **f** to **j** showed the satisfactory functional outcomes at the last visit at 14 months postoperatively

Figures 1 and 2 presented 2 typical cases of scaphoid waist fracture nonunion treated by the tripod fixation technique (Fig. 1) or the single Herbert screw fixation (Fig. 2). Bony union was achieved at 15 weeks and 17 weeks, respectively. At the last visit, the ROM showed satisfactory results.

## Discussion

For most scaphoid fracture nonunion cases, stable internal fixation of autogenous bone graft via cannulated compression screws remains the gold standard regimen, but the inadequate anti-rotation ability may be a potential issue. In this study, we used the “tripod” technique, namely one headless compression screw and two anti-rotational K-wires, for treatment of unstable scaphoid waist nonunion. The results showed the trend towards higher bony union rate (100% vs 95.4%, *p* = 0.122) and the significantly less time to union (14.8 vs 17.6 weeks, *p* = 0.027) in the tripod fixation group than in the single cannulated screw fixation group. With respect to clinical and radiographic outcomes and the overall rate of complications, no significant difference was found. This suggested that tripod technique can facilitate and accelerate the bone union, while having the similar clinical effectiveness and safety.

The use of multiple K-wires for treatment of the scaphoid nonunion is common, due to its superiority in bony union. In a most recent study by Hegazy et al [15], three convergent 1.1-mm K-wires were placed in retrograde manner for stabilizing scaphoid fracture nonunion, and the union rate was 98% in the K-wire group vs 89% in the Herbert screw group, as well as the shorter union time (12 vs 15 weeks). Chen et al [16] used divergent K-wires to stabilize the reduced scaphoid fragments in 26 patients with scaphoid nonunion, and the 3 to 6-year follow-up assessments showed the 100% union rate within postoperative 4 months and the good or excellent functional outcomes in all patients. Meisel et al [17] retrospectively reviewed one single senior surgeon's experience of 32 cases of scaphoid nonunion treated by iliac crest bone graft and convergent K-wire fixation, and reported the 100% of union rate and 17.9 weeks to achieve bony union. The explanation could be the better control for rotation of fragments with use of multiple K-wires, relative to a single screw. The biomechanical or finite element analysis have also demonstrated this inference [18, 19]. However, in some selected cases where the fragments were fragile, small or flat, K-wire fixation is not an optimal but a fallback option [8].

It should be noted that, use of multiple K-wires alone may not adequately resist the bending forces when fingers are moving to generate the axial compression force at the fracture site. This can partially explain why the long immobilization period was needed in some studies [15, 16]. On the other hand, relative to K-wires, a single cannulated screw has superiority in resisting bending force, but may not adequately withstand cyclical rotation or multi-axis loading [18, 20]. In this study, we combined the advantages of both fixation methods that use a cannulated screw and the two additional K-wires to form a “tripod” structure, which potentially maximized the anti-bending and the anti-rotation ability to maintain the fragments to union. We thought this was the major reason that the tripod fixation achieved 100% rate of union and the significantly shorter time to union, as compared to the single screw fixation group.

The choice of bone graft will also have effect on the union of fragments. In literature, the cancellous iliac bone graft was often a preferable choice. Relative to cortico-cancellous bone graft harvested from the distal radius, the cancellous iliac bone graft has a thicker trabecular architecture and higher concentration of osteoblasts and osteocytes, thus providing a greater osteogenic potential and allowing quicker revascularization and incorporation at the nonunion site [21, 22]. However, in some clinical studies where the same fixation techniques were used, no difference was observed between the choices of bone grafts [23, 24]. We suggest that scaphoid union depend on the fixation stability more than the choice of bone graft, and if given the stable circumstances, the high union and accelerated union is predictable.

There were several limitations to this study. Firstly, the retrospective design was its inherent limitation. This would have caused the selection bias due to the non-randomization, despite non-significant differences observed for the demographics and preoperative variables. Secondly, we could not capture the patient’s preoperative radiographic parameters or functional status, which would have affected the wrist functional recovery and the comparative results between two methods. Thirdly, the sample size was small due to the scarcity of such complication, which is susceptible to a type I statistical error. However, we have tried the best to retrieve the medical records from a relatively long study period, and in fact, the sample size herein was comparable to that in most previous studies. Fourthly, this was a single-center study in a tertiary referral center, where more complex or severer cases were transferred. Therefore, our results might be less generalizable to other settings, but our pre-defined scaphoid fracture nonunion in waist would partially compensate for this bias.

In conclusion, the open reduction, cortico-cancellous bone grafting and fixation with tripod technique (headless compression screw and two K-wires) is an effective and safe alternative to the traditional one single Herbert screw fixation. This technique can be considered to use in clinical practice, especially for those potentially rotationally unstable scaphoid nonunion cases.

## Data Availability

All the data will be available upon motivated request to the corresponding author of the present paper.

## Abbreviations

ROM: Range of motion
VAS: Visual analogue scale
DASH: Disabilities of the arm, shoulder and hand
SNAC: Scaphoid nonunion advanced collapse
AVN: Avascular necrosis
FCR: Flexor carpi radialis tendon
LISA: Lateral intrascaphoid angle
HLR: Height length ration
SD: Standard deviation
